# The Life Quality of Rheumatoid Arthritis Patients Seeking Spa Radon Therapy: A Pilot Study

**Published:** 2019-12

**Authors:** Jaroslav STANCIAK, Monika MACKINOVA, Martin SAMOHYL

**Affiliations:** 1. Department of Social Work, Faculty of Education, Comenius University in Bratislava, Bratislava, Slovak Republic; 2. Institute of Hygiene, Faculty of Medicine, Comenius University in Bratislava, Bratislava, Slovak Republic

## Dear Editor-in-Chief

Rheumatoid arthritis is a progressive autoimmune disease that causes stiffness of joint, pain, and swelling (1, 2). The incidence of rheumatoid arthritis (per 1,000 inhabitants) in Slovak population was 0.1–0.2 in 2008 (for men) and 0.2–0.4 (for women) (3). “The arthritis patient–medical encounters will be effective when public health efforts of arthritis are successful” (4).

The aim of the study was to analyze the spa radon therapy effect in rheumatoid arthritis patients. In the study sample were rheumatoid arthritis patients (n=50) treated in Radon spas (21 days) in the Czech Republic.

The standardized questionnaire was collected from 2013 to 2016. *The mean age* of the rheumatoid arthritis *patients was* 62.1 years.

In this pilot study, the standardized health-related quality of life questionnaire was used (5). The statements in the survey instrument are divided into eleven subscales: physical functioning, role physical, bodily pain, general health, vitality, social functioning, role emotional, mental health, overall physical health, overall mental health and overall quality of life. The questions in standardized questionnaire were evaluated in scale 0–100 (100=the highest level of quality of life).

Rheumatoid arthritis patients (males and females) were significantly more satisfied with quality of life after spa radon therapy in subscales: role physical (*P*<0.001), bodily pain (*P*<0.05), vitality (*P*<0.05), role emotional (*P*<0.001), overall physical health (*P*<0.05), overall mental health (*P*<0.05) and overall quality of life (*P*<0.05) (Table 1). The positive effect of spa radon therapy was confirmed.

**Table 1:** Quality of life in rheumatic arthritis *patients* before and after spa therapy according to gender (n=50)

***Subscales***	***Mean score x***			
	***Males (n = 25)***		***Females (n = 25)***	
	***Before a spa therapy***	***After a spa therapy***	***Before a spa therapy***	***After a spa therapy***
Physical Functioning	66.7	72.1	60.8	67.9
Role Physical	36.2	52.4^**^	36.2	51.2^**^
Bodily Pain	38.1	55.7^*^	41.2	54.7^*^
General Health	36.5	46.2	40.8	48.2
Vitality	48.2	61.2^*^	49.3	59.3^*^
Social Functioning	59.1	62.3	51.7	61.3
Role Emotional	61.8	82.5^**^	53.2	70.5^**^
Mental Health	62.8	68.2	55.8	62.3
Overall Physical Health	44.4	56.6^*^	44.8	55.5^*^
Overall Mental Health	58.0	68.6^*^	52.5	63.4^*^
Overall Quality of Life	51.2	62.6^*^	48.6	59.4^*^

* *P*<0.05 //

* P<0.001
